# Skewed risk perceptions in pregnant women: the case of influenza vaccination

**DOI:** 10.1186/s12889-015-2621-5

**Published:** 2015-12-29

**Authors:** Birte Bödeker, Cornelia Betsch, Ole Wichmann

**Affiliations:** Department for Infectious Disease Epidemiology, Immunization Unit, Robert Koch Institute, Seestraße 10, 13353 Berlin, Germany; Charité—University Medicine Berlin, Augustenburger Platz 1, 13353 Berlin, Germany; Department of Psychology, Center for Empirical Research in Economics and Behavioral Sciences, University of Erfurt, Nordhäuserstraße 63, 99089 Erfurt, Germany

**Keywords:** Influenza, Vaccination, Pregnancy, Attitudes, Risk perception, Germany

## Abstract

**Background:**

Pregnant women and their newborns have an increased risk of developing severe influenza and influenza-related complications. In Germany, seasonal influenza vaccination is recommended for pregnant women since 2010. However, little is known about pregnant women’s vaccination-related knowledge and attitudes, as well as their risk perceptions. This study therefore assessed pregnant women’s vaccination-related knowledge, risk perceptions related to influenza disease and influenza vaccination during pregnancy, and aimed to identify determinants of influenza vaccination uptake during pregnancy in Germany.

**Methods:**

Between 2012 and 2014, a nationwide web-based prospective cohort study with follow-up interviews was conducted in initially pregnant women who gave birth over the study period. Control groups were set up in a cross-sectional fashion during the follow-up interviews. Women who participated in both, the baseline interview before giving birth and in the 1st interview after giving birth were included in the analysis. Univariate and multiple logistic regression were used to identify associations between influenza vaccination uptake and sociodemographic characteristics as well as items assessing attitude and knowledge.

**Results:**

In total, 838 women were included in the analyses. Pregnant women had a positive attitude towards vaccination in general, but only modest vaccination knowledge. Overall, 10.9 % of women were vaccinated against seasonal influenza during pregnancy. While pregnant women perceived classical childhood diseases to be more risky than the respective vaccinations, this relation reversed for influenza: The risk of vaccination was perceived higher than the risk of the disease. These two types of risk perceptions independently determined influenza vaccination uptake—higher perception of disease risk and lower perceptions of vaccination-related risks increased uptake. Additionally, knowledge about the vaccination recommendation for pregnant women and a positive gynaecologist’s attitude towards vaccination during pregnancy influenced the uptake significantly.

**Conclusions:**

Influenza vaccination uptake in pregnant women is low in Germany. Tailored communication strategies for pregnant women should focus especially on changing the perceptions of personal risks regarding influenza and influenza vaccination during pregnancy. Gynaecologists should be made aware about their crucial role in supporting vaccination decision-making of pregnant women and the need to provide relevant information to counteract misconceptions.

## Background

Pregnant women and their newborns have an increased risk of developing severe influenza and influenza-related complications [1–3]. Influenza during pregnancy can cause stillbirth, preterm delivery, and growth retardation in the child [4, 5]. Therefore, the World Health Organization and National Immunization Technical Advisory Groups in many industrialized countries recommend seasonal influenza vaccination for pregnant women [6–8].

In Germany, seasonal influenza vaccination is recommended for all pregnant women from the 2nd trimester and for pregnant women with underlying chronic disease from the 1st trimester since August 2010 [9]. There is no other vaccination that is officially recommended during pregnancy in Germany as of today.

Similar to other European countries [7], a cross-sectional study in Germany showed low (16 %) seasonal influenza vaccine uptake among pregnant women for the 2012/13 influenza season [10]. Major reasons for being unvaccinated were a lack of confidence in the vaccine and the perception that vaccination was not necessary. Beyond these reasons, it is important to also assess the predictive validity of risk perceptions as well as attitudes and vaccine-related knowledge, as these have proven influential determinants of the individual vaccination decision (overview see [11]). Such data are crucial for developing tailored communication strategies to improve influenza vaccination coverage in this at-risk group [12]. However, too little is currently known about pregnant women’s attitudes and perceptions related to vaccination during pregnancy in Germany.

The present study aimed at closing this gap. Between 2012 and 2014 we carried out a nationwide prospective cohort study in primigravida pregnant women who gave birth over the study period, with cross-sectional control groups at follow-up interviews. The longitudinal part of the study aimed at assessing changes in vaccination-related behaviour, attitudes, knowledge and risk perceptions that could occur from pregnancy to early motherhood. Here we present results from a sub-analysis on the risk perception and attitudes related to influenza and seasonal influenza vaccination during pregnancy. Specifically, this sub-analysis had the objectives to (i) assess knowledge related to vaccinations in general, (ii) compare risk perceptions related to influenza vaccination during pregnancy with risk perceptions related to classical childhood vaccinations from a pregnant woman’s perspective, (iii) estimate influenza vaccination coverage in pregnant women in 2011/12 and 2012/13, (iv) investigate reasons for not being vaccinated against seasonal influenza, and (v) identify further determinants of influenza vaccination uptake during pregnancy.

## Methods

### Study design and population

Between February 2012 and August 2014, we conducted a nationwide web-based prospective cohort study in initially pregnant women who gave birth over the study period. We collected data at recruitment during pregnancy, and followed up on this three more times until the child was 14 months. To identify possible learning effects through study participation, control groups were set up in a cross-sectional fashion during the follow-up interviews. Those belonging to the longitudinal group were surveyed 3 more times after giving birth to their child (+3, +6, and +14 months after childbirth), whereas women of the control groups were surveyed only once again (+3, +6, or +14 months after childbirth). The timing of the follow-up interviews was based on the vaccination schedule of the German Standing Committee of Vaccination (STIKO) [13]. Inclusion criteria for study participation (both for women in the longitudinal group and the cross-sectional control groups) were (i) being at least 18 years of age, (ii) pregnant, (iii) primigravida, and (iv) living in Germany.

**Fig. 1:**
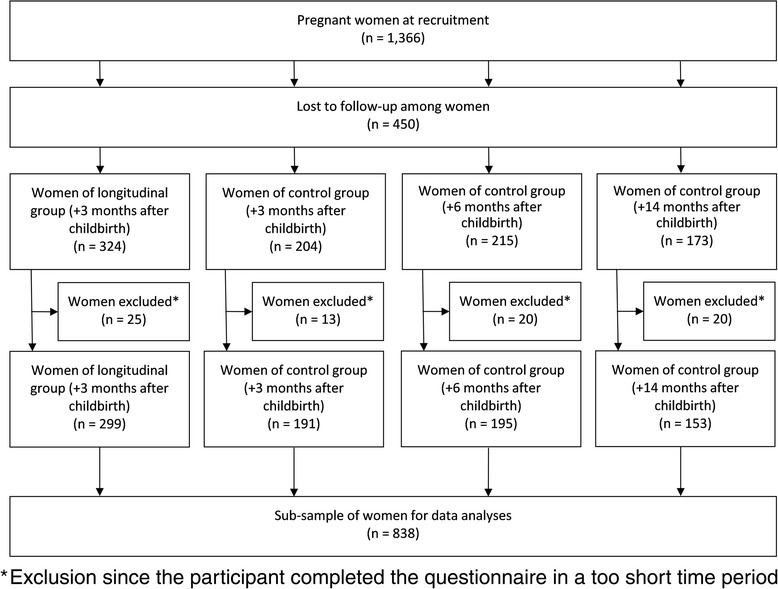
Flowchart indicating the number of total participants and their assignment into different subgroups

At study entry (between February and August 2012), all pregnant women completed an online study questionnaire and were subsequently randomly assigned to either the longitudinal group or to one of the three control groups. For this sub-analysis we included women who participated both at study entry before giving birth and in the 1st interview after giving birth, where questions related to influenza and influenza-vaccination were asked (either +3, +6 or +14 months after giving birth; see Fig. 1). Participants who completed the questionnaires in a very short time (defined as <360 s) were excluded to improve data quality. Due to the length of the questionnaire this time-limit was estimated to be the minimum time for serious participation. Women who took part in the study twice were identified by cross-checking the personal codes as well as email addresses for doublets to reduce the possibility of repeat participation. Datasets with the same codes or email addresses were both eliminated from the data set.

Study participants were recruited via different websites and information portals targeting pregnant women. As an incentive for study participation, at recruitment women were enrolled in an online lottery draw for vouchers worth €20 each. Additionally, women belonging to the longitudinal group received vouchers worth €10 for participation in follow-up interview at +3 months after childbirth and women of the control group took part in a lottery draw for vouchers worth €20 each. To register for the study, women were asked to provide their email address that was only used for invitation and reminders (up to 2 times) to participate in the study as well as for the lottery draw and distribution of vouchers.

According to a sample size calculation, 843 pregnant women were needed to detect possible differences of at most 3 % from an assumed vaccination coverage of 10 % (9 % were vaccinated against pandemic influenza in 2009/10 in this target group [14]) with a confidence interval of 95 % and a power of 80 %. Since seasonal influenza vaccination uptake during pregnancy was not surveyed at the time of recruitment but during the first interview after giving birth (i.e. either +3, +6 or +14 months after giving birth), we considered a possible dropout of approximately 30 % leading to 1,200 pregnant women who should be recruited.

### Questionnaire

We used structured online questionnaires to collect information on (i) general vaccination-related attitude and knowledge, prior experiences with vaccinations as well as the preference for conventional or alternative medicine, (ii) risk perceptions of vaccine-preventable diseases and the respective vaccinations, (iii) behaviour, attitudes and knowledge related to influenza disease and seasonal influenza vaccination, and (iv) sociodemographic factors. Items of (i), (ii) and (iv) were asked at recruitment and items of (iii) were surveyed at the first follow-up interview after recruitment. All questions were self-reported and not validated.

#### Attitude towards vaccination and general vaccination knowledge

For our analysis, independent from belonging to the longitudinal or control group, all the following information were collected at recruitment: Participants rated their general vaccination-related attitude and experience on a scale from 1 (“totally against vaccination” and “very negative”, respectively) to 100 (“totally in favour of vaccination” and “very positive”, respectively). General knowledge about vaccination was assessed using the knowledge test developed by Zingg and Siegrist [15], addressing the most prevalent misconceptions about vaccination. The scale comprises 9 true-false statements. Women rated all statements as “true”, “false”, or “don’t know”. General knowledge was calculated as the sum score of all correct answers (range 0–9). “Don’t know” answers were defined as wrong responses [15]. The higher the score, the greater the correct knowledge is. To measure the general preference for conventional or alternative medicine, women were asked on a scale from 1 to 100 whether they would rather consult an alternative practitioner or a conventional doctor if being sick (“1” consulting an alternative practitioner vs. “100” consulting a doctor). Moreover, participants rated their gynaecologist‘s attitude towards vaccination during pregnancy and their midwife’s attitude towards vaccination on a scale from 1 (“contra vaccination”) to 100 (“pro vaccination”). In contrast to the previous information, these two items were collected at the first follow-up interview after recruitment.

#### Risk perception

Risk perception was assessed at recruitment for the following vaccine-preventable diseases and the respective vaccinations: (i) seasonal influenza for pregnant women, and (ii) varicella, measles, pneumococcal disease (described as responsible for pneumonia), tetanus, pertussis, and hepatitis B infection for the child. To assess risk perceptions, women rated their perceived probability of acquiring the disease and probability of side effects following vaccination (range “0” to “100 %”), as well as for the perceived severity of the disease and perceived severity of vaccination side effects (range 1 to 100: “not serious” to “very serious”). Since perceived risk is a function of the perceived probability of an event and its expected consequences, we measured the perceived risk as the mathematical product of the perceived probability of acquiring the disease and perceived disease severity [16]. After multiplication we fit the ranges to 0.01 to 100. While the risk assessment of influenza referred to the disease and vaccination during pregnancy, all other diseases and vaccinations referred to their future child.

#### Influenza vaccination

All women answered the following influenza vaccination-related questions at the first follow-up after recruitment, which was 3+ months after giving birth for the longitudinal group and at either 3+, 6+ or 14+ months after giving birth for the control groups. Influenza vaccination uptake was defined as having received a flu shot during pregnancy. Unvaccinated women were asked to express their reasons for not getting vaccinated by selecting from a predefined set of possible answers (multiple answers possible). Furthermore, knowledge of STIKO vaccination recommendations during pregnancy was surveyed. Women were asked to state which of the following vaccinations (tetanus, influenza, rotavirus, hepatitis B) were recommended by the STIKO for pregnant women. After providing their responses, participants were provided with the correct answer (influenza).

#### Sample characteristics

For sociodemographic factors, at recruitment we collected data on age, country of birth, place of residence, education level, employment status, and monthly household income. Pregnancy trimester at the time of recruitment was calculated based on the date of recruitment and the expected date of birth.

### Statistical analysis

We performed descriptive statistics to describe the study population, general vaccination-related knowledge, risk perceptions among pregnant women, and reasons for not being vaccinated against influenza during pregnancy. To analyse the association between vaccination-related knowledge and sociodemographic variables we calculated Spearman correlation coefficient. To determine potential associations between influenza vaccination uptake and sociodemographic characteristics as well as attitude and knowledge items, we conducted univariate and multiple logistic regression analyses. Odds ratios (OR) and 95 % confidence intervals (CI) were calculated. A *p*-value of <0.05 was considered statistically significant. Variables with a *p*-value of <0.1 in the univariate analysis were entered in the first step of the multiple analyses. We then removed non-significant factors (≥0.05) from the model in a stepwise backward procedure to obtain the final model. Missing data were not replaced or imputed. Statistical analyses were performed with StataSE13 (StataCorp LP, College Station, TX, USA).

### Ethical considerations and data protection

Participants were informed about study details, including data protection and privacy issues. Participation in the study was only possible after the women provided an informed consent via the online questionnaire. All data were collected and analysed anonymously. Emails for the lottery draw and distribution of vouchers were technically separated from the data of the questionnaire. The study was approved by the ethics committee of Charité University Medicine, Berlin (Charité, EA1/010/12).

## Results

### Recruitment and sample characteristics

In total 1,366 pregnant women qualified for the study and completed the recruitment questionnaire. Of these, 916 women also participated in the 1st interview after giving birth, where questions related to influenza and influenza-vaccination were asked. Since 78 participants completed the questionnaires in a very short time, they were excluded from the dataset, resulting in 838 women included in the final analyses (Fig. 1). All surveyed women were pregnant during influenza seasons in 2011/12 or 2012/13. An overview of participants’ characteristics compared to the general female population aged 18–49 years living in Germany is shown in Table 1. The median age of participants was 30 years (range 18–42). More than three quarters of women had an university entrance diploma and more than half of women were at the time of study enrolment in their 3rd trimester of pregnancy.

**Table 1:** Characteristics of study population at recruitment and the general female population aged 18–49 years living in Germany in 2012

Characteristics	Study population, % (95 % CI)	Genereral female population, % (*n* = 16,573,000)^a^
Age-group (*n* = 836)		
18–24 years	9.0	18.4
25–29 years	33.7	14.4
30–34 years	41.6	14.7
35–39 years	14.4	18.3
40–49 years	1.3	20.3
Country of birth (*n* = 838)		
Germany	94.8	82.2
Other country	5.3	17.8
Place of residence^b^ (*n* = 838)		
Eastern Federal States	27.2	18.7
Western Federal States	72.8	81.3
Education level^c^ (*n* = 838)		
Low	1.3	22.0
Middle	15.5	37.5
High	83.2	38.2
Employment (*n* = 837)		
Not employed	16.1	25.7
Part-time employed	11.8	32.2
Full-time employed	72.0	42.1
Monthly household income (*n* = 826)		
≤ 1500 €	10.4	18.4
1501–2000 €	11.9	11.1
2001–2500 €	14.3	59.2
2501–3000 €	17.1	
≥ 3001 €	46.4	
Pregnancy trimester (*n* = 836)		
First	13.3	--
Second	29.8	--
Third	56.9	--

^a^Data from the microcensus 2012 from the Federal Statistical Office of Germany [48]. Since data concerning household income and education level was not available for each women, data cannot result in 100 % ; ^b^Eastern Federal States: Mecklenburg-Vorpommern, Brandenburg, Berlin, Saxony, Saxony-Anhalt, Thuringia; Western Federal States: Schleswig-Holstein, Bremen, Hamburg, Lower Saxony, Hesse, Rhineland-Palatinate, Saarland, North Rhine-Westphalia, Bavaria, Baden-Württemberg; ^c^Low: nine years or less of school education, middle: at least 10 years of school education, high: university entrance diploma

### Attitude towards vaccination in general

Overall, pregnant women had a positive general vaccination-related attitude (mean: 81.5, 95 % CI 80.0–83.0). However, women rated their gynaecologist’s attitude towards vaccination during pregnancy (mean: 66.0, 95 % CI 63.6–68.4) and their midwife’s attitude towards vaccination in general as rather moderate (mean: 60.8, 95 % CI 58.5–63.1).

### General vaccination knowledge

Participants’ answers to the items of the vaccination knowledge test are presented in Table 2. Overall, general vaccination knowledge was moderate in pregnant women. Half of them stated at least six correct answers (median: 6; range: 0–9; mean: 5.8, 95 % CI 5.6–5.9). Almost all pregnant women knew that vaccinations are not superfluous. The majority also stated that the efficacy of vaccines has been proven and that massive vaccination programs are important to eliminate specific diseases.

**Table 2:** General vaccination knowledge of 838 pregnant women, Germany 2012

Item	Statement	Response [%]		
		Correct	Incorrect	Don’t know
1	The additives used in vaccines are not dangerous for humans (true)	43.4	23.2	33.4
2	Diseases like autism, multiple sclerosis, and diabetes might be triggered by vaccinations (false)	58.2	7.5	34.3
3	Vaccinations increase the occurrence of allergies (false)	58.1	12.1	29.8
4	Vaccines are superfluous, as diseases can be treated, e.g. with antibiotics (false)	94.8	1.8	3.5
5	Without massive vaccination programs, smallpox would still exist (true)	81.0	4.4	14.6
6	The efficacy of vaccines has been proven (true)	87.7	4.5	7.8
7	Children would be more resistant if they were not always vaccinated against all diseases (false)	63.6	16.0	20.4
8	Many vaccinations are administered too early. As a result, the body’s own immune system has no possibility to develop by itself (false)	45.5	20.2	34.4
9	The immune system of children will not be overwhelmed by a high number of vaccines (true)	44.9	15.5	39.6

Knowledge test developed by [15]

However, many pregnant women were unsure in responding to other items, leading to a high percentage of participants quoting “don’t know” as an answer. For example, 40 % of women did not know that the immune system of children will not be overwhelmed by a high number of vaccines. Moreover, almost one quarter of participants believed that additives used in vaccines are dangerous and that many vaccinations are administered too early.

Age and education level were significantly associated with knowledge (r = .10, *p* < 0.05 for both variables), indicating that pregnant women with an increased age and with higher education level had a better general knowledge about vaccination. There were no significant relations to other socio-demographic variables.

### Risk perception

**Fig. 2:**
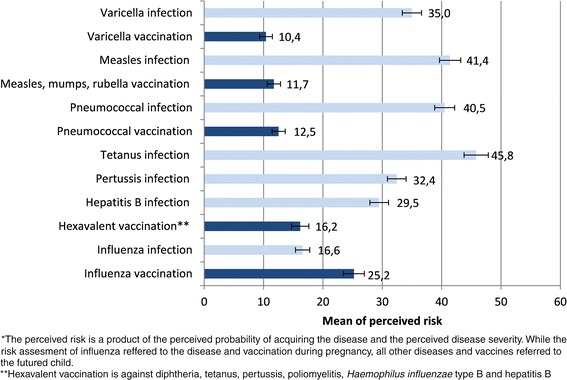
Perceived risk* of vaccine-preventable diseases and the corresponding vaccinations among pregnant women, Germany, 2012

Figure 2 shows the means of perceived risks of vaccine-preventable diseases and the respective vaccinations among pregnant women. The highest disease-specific risks were considered for an infection with tetanus, measles and pneumococcal disease for children in their lifetime. Varicella vaccination was assigned the lowest perceived risk compared to other vaccinations. For children the perceived risk of the disease was always rated significantly higher than the perceived risk of the vaccination. However, this pattern reversed for perceptions related to influenza vaccination during pregnancy: influenza vaccination during pregnancy was perceived significantly more risky than acquiring an influenza virus infection during pregnancy.

### Influenza vaccination and reasons for not being immunized

Influenza vaccination status was available for almost all participants (99.6 %). Overall, 91 women (10.9, 95 % CI 8.9–13.2) stated that they had received a seasonal influenza vaccination during pregnancy: 18 (19.8 %) in their 1st, 47 (51.7 %) in their 2nd and 26 (28.6 %) in their 3rd trimester of pregnancy.

**Fig. 3:**
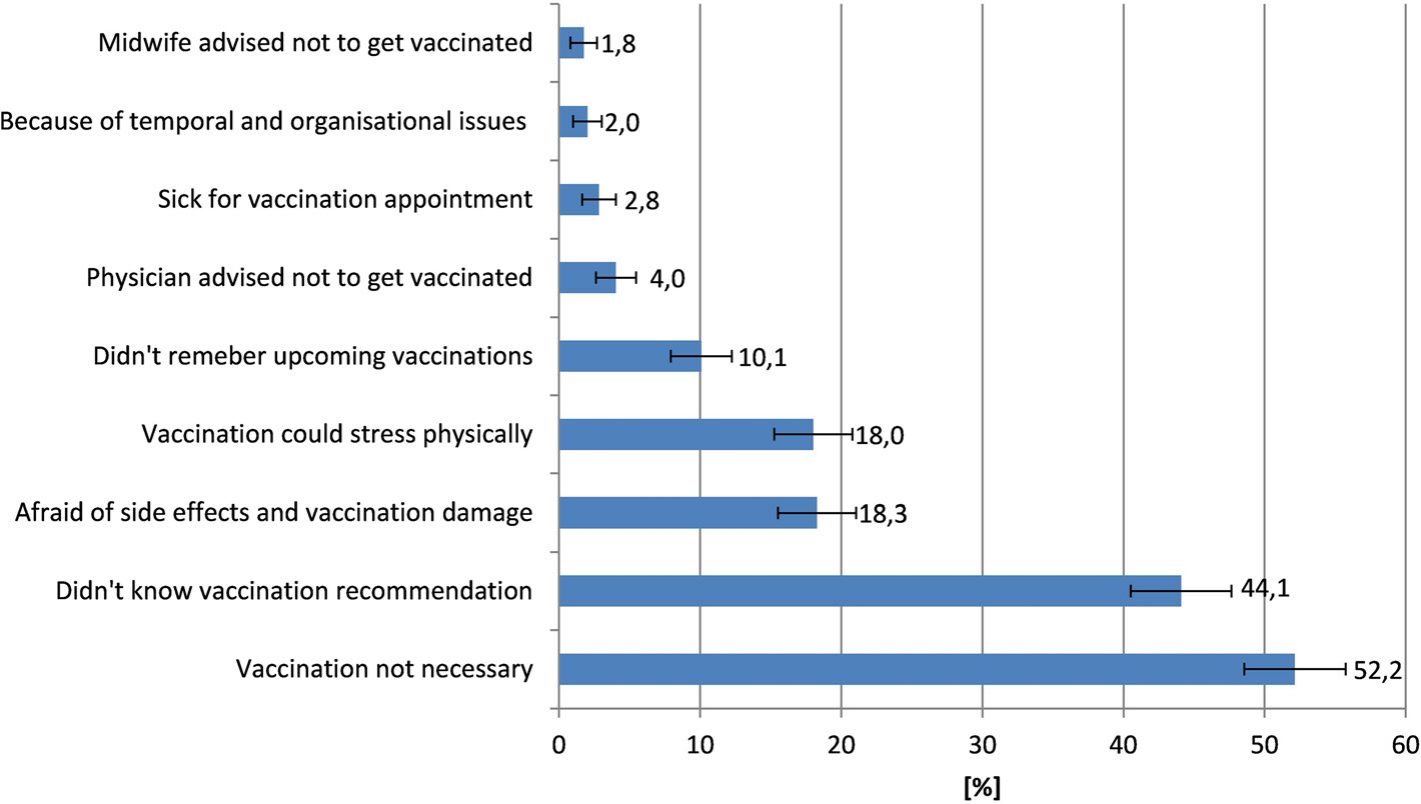
Reasons against seasonal influenza vaccination stated by 744 unvaccinated women, Germany (multiple answers were allowed)

The most frequently stated reasons for not having received an influenza vaccination were the perception that vaccination is not necessary, lacking awareness of the influenza vaccination recommendation for pregnant women, and mistrust in the vaccine (Fig. 3). Among unvaccinated women, those who did not know the vaccination recommendation stated in 37.2 % that the vaccination is not necessary, whereas those who did not mention absent knowledge of the vaccination recommendation as a reason for being unvaccinated stated in 63.9 % that vaccination is not necessary (p < 0.001). Independent of their vaccination status, about half of all participants (54.4, 95 % CI 51.0–57.8) were aware and 45.6 % (95 % CI 42.2–49.0) were not aware of the STIKO recommendation in respect to vaccination of pregnant women against seasonal influenza. However, many women incorrectly thought that STIKO recommends pregnant women also vaccination against tetanus (38.9, 95 % CI 35.6–42.3), hepatitis B (33.8, 95 % CI 30.6–37.1), hepatitis A (21.7, 95 % CI 19.0–24.7), and rotavirus (11.5, 95 % CI 9.4–13.8). When asking vaccinated women how they were advised of the influenza vaccination, almost all participants mentioned their physician (90.1, 95 % CI 82.1–95.4) followed by family and friends (17.6, 95 % CI 10.4–27.0). The majority of vaccinated women stated their physician as an important source of information about influenza vaccination during pregnancy (85.7, 95 % CI 76.8–92.2), followed by the Internet (23.1, 95 % CI 14.9–33.1) and family and friends (13.2, 95 % CI 7.0–21.9). Print media was only mentioned by 6.6 % (95 % CI 2.5–13.8).

### Factors associated with influenza vaccination uptake

Table 3 presents the results of univariate and multiple logistic regression analyses of factors influencing influenza vaccination uptake in pregnant women. Influenza vaccination uptake was independently associated with the knowledge of the influenza vaccination recommendation for pregnant women, a higher perceived gynaecologist’s attitude towards vaccination during pregnancy, and an increased perceived risk of influenza infection; it was negatively associated with an increased perceived risk of influenza vaccination. Knowledge and attitudes related to vaccination in general were not independently associated with influenza vaccination uptake.

**Table 3:** Factors potentially associated with influenza vaccination uptake during pregnancy, Germany

Factor	Vaccination coverage, %^a^	Univariate OR (95 % CI)^a^	Multiple OR (95 % CI)^b^
Age-group			
18–24 years	4.0	Ref.	NS
25–29 years	12.8	3.53 (1.06–11.79)	
30–34 years	10.7	2.87 (0.86–9.58)	
35–39 years	11.7	3.17 (0.88–11.43)	
40–49 years	9.1	2.4 (0.23–25.36)	
Knowledge of STIKO-recommendation during pregnancy			
Yes	18.2	10.38 (4.95–21.74)	8.0 (3.35–19.12)
No	2.1	Ref.	Ref.
Preference for conventional or alternative medicine	−	1.02 (1.00–1.03)	NS
Prior experiences with vaccinations	–	1.02 (1.01–1.03)	NS
General vaccination knowledge	–	1.32 (1.18–1.48)	NS
General vaccination-related attitude	–	1.03 (1.02–1.05)	NS
Gynaecologist’s attitude towards vaccination during pregnancy	–	1.04 (1.03–1.06)	1.04 (1.03–1.06)
Midwife’s attitude towards vaccination in general	–	1.01 (1.00–1.02)	NS
Perceived risk of influenza infection	–	1.04 (1.03–1.05)	1.05 (1.03–1.06)
Perceived risk of influenza vaccination	–	0.96 (0.95–0.97)	0.97 (0.96–0.98)
BIC			−128.84
Cragg & Uhler's R2			0.45

Other nonsignificant variables in univariate analysis (*p* ≥ 0.1) were: country of birth, place of residence, education, number of screening examination during pregnancy

*NS* not significant; *Ref* reference category

^a^Included participants with information on relevant item; ^b^Included *n* = 495 participants with complete information on all items

## Discussion

Pregnant women are at increased risk for severe influenza and constitute a target group for seasonal influenza vaccination in Germany since 2010. In our study population, vaccination uptake of 11 % during the 2011/12 and 2012/13 influenza season was suboptimal, which calls for an enhancement of activities to improve influenza vaccination coverage in this at-risk group in Germany. Among study participants, women possessed a medium level of vaccine-related knowledge and had a positive attitude towards vaccination in general. The perceived risks of childhood diseases were always rated higher than the perceived risk related to the respective vaccine. However, for influenza the perceived risk of suffering from side effects after influenza vaccination during pregnancy was higher than of contracting influenza.

In a recently published cross-section survey conducted in Germany during the 2012/13 influenza season, an equally low seasonal influenza vaccination coverage (16, 95 % CI 13.7–18.4) was identified among pregnant women [10]. In that study, a different study design and participant recruitment was applied (i.e. cross-sectional study where pregnant women were recruited based on a special sampling frame [ADM-Sampling-System for Face-to-Face Surveys] designed as an area sample), which allowed the assembly of a representative sample for Germany. A recent published review summarized vaccination rates among pregnant women in various industrialized countries ranging from 2 to 88 % [17]. These substantial differences between countries might be explained by different communication activities supporting the vaccination recommendations, differences in vaccination systems and funding schemes, and also in different attitudes related to seasonal influenza vaccination.

In our study we were able to show that the knowledge of the influenza vaccination recommendation during pregnancy, the vaccination-related attitude of the gynaecologists, and risk perceptions play a major role in the pregnant women’s influenza vaccination decision-making process. Findings of other studies revealed that health care providers were more likely to recommend vaccination if they have a positive attitude towards influenza vaccination during pregnancy [18–20]. This finding was confirmed in a study among occupational physicians in Germany, where an association between a positive attitude towards vaccination and a higher likelihood of recommending influenza vaccination to pregnant women was identified [21]. According to the review of Yuen et al. [17], pregnant women were 20 to 100 times more likely to receive the flu shot when being advised of the vaccination by a health care provider. We found that in addition to physicians the Internet was also mentioned as an important source of information about influenza vaccination. However, vaccine-related information from the Internet must be considered with caution since misconceptions about vaccination are prevalent and could influence the perceived risk of vaccination and the intention to vaccinate [22, 23].

In our study, the most commonly stated reasons against having received a flu shot were the absent knowledge of the vaccination recommendation but also a perception of being at low risk for acquiring influenza. This indicates that communication strategies will fall short if only focusing on making pregnant women acquainted with the existence of the recommendation. In fact, there is a need for high-quality patient education to make women aware of the importance of vaccination and the safety of flu vaccines during pregnancy. Other studies also indicated that pregnant women were insufficiently informed about the risk of influenza during pregnancy and the benefits of vaccination [10, 17, 24–26]. A study from the US found for example that only 23 % of pregnant women compared to 35 % of non-pregnant women stated being at risk for vaccine-preventable diseases [27]. When rating the perceived risks of different diseases and the respective vaccinations, our findings showed the highest perceived risk for infections that refer to children. Among those diseases, pregnant women perceived the lowest risks for hepatits B, pertussis and varicella. This perception is also reflected in the national vaccination coverage estimates from school entrance examinations in Germany, that show a lower uptake of hepatitis B and varicella vaccines when compared to other vaccines such as measles, tetanus, pertussis or polio [28].

As a major finding, we identified a reversed risk perception regarding the infection with influenza and influenza vaccination during pregnancy, leading to a higher risk perception of the vaccination compared to the disease. The lack of an appropriate influenza risk perception among pregnant women is alarming and must be considered in tailoring communication strategies that ideally are directed at the factors that impede vaccination [12]. The results of our study demonstrate that it is necessary to focus on changing the perceptions of personal risks regarding the disease and the vaccination during pregnancy [11]. Independent of the vaccination and population, studies showed that risk perception is a main predictor of vaccination behaviour and that immunization decisions are strongly influenced by emotional factors [29–34]. In this respect, we were also not able to find any association between general vaccination knowledge and influenza vaccination uptake in pregnant women. Our data suggest that in the case of influenza and pregnant women it is the specific knowledge about vaccination during pregnancy that relates to the vaccination decision. Also the general attitude towards vaccination did not influence the influenza vaccination decision. We assume that this attitude is a result of an evolving process that is formed particularly after making the first vaccination experiences with the own child, acting on the assumption that the general vaccination attitude of mothers and pregnant women normally is unimpaired and overall good. Similar to our results, a Canadian study among mothers of young children indicated a generally positive attitude of vaccinations [35]. In a European project, parents with children less than 3 years of age had generally positive attitudes towards immunizations in the childhood vaccination programs, and between 81 and 97 % of parents would immunize their child in the future [36]. In further investigations that base on the data of the presented study we will be able to analyse if the attitude towards vaccination changes over time and which events or sources of information instigate and influence changes in attitudes. For pregnant women, several studies have demonstrated that the majority is willing to get vaccinated during pregnancy if recommended by their health care provider [27, 37, 38]. In respect to the influenza vaccination, pregnant women must be educated more specifically about the benefits and risks of influenza vaccination in pregnancy not only with a focus on protecting themselves but also their child. These findings should be considered in the national information campaign that is conducted annually in Germany to raise awareness about the importance of influenza vaccination and to increase vaccination uptake in the main target groups including pregnant women.

Of concern, we found overall moderate vaccination knowledge in pregnant women. Interventions targeted at pregnant women should therefore aim at improving influenza specific and general vaccination-related knowledge. Studies from Spain and Germany also identified several prevalent vaccination-related misconceptions in parents [39, 40]. It was also shown that the immunization status of children was influenced by vaccination knowledge of pregnant women and parents [15, 39, 41]. Thus, pregnancy provides a unique opportunity to educate future parents about immunization. Other study findings revealed that the vaccine decision-making process begins prenatally and is an evolving process [29, 42, 43]. Wroe et al. [29] demonstrated that 88 % of women made the immunization decision for their child antenatally. Parents of a study conducted in England even suggested receiving information prior to childbirth to make more informed vaccination decisions [42]. Therefore, the perinatal period represents an important time for providing education about immunizations. Especially gynaecologists should use the frequent check-ups during antenatal care for educating pregnant women on the importance of vaccinations. In Germany, based on a nationwide standardized program of prenatal care, each pregnant woman receives an official booklet at the beginning of pregnancy that comprises all medical check-ups and their results during pregnancy. However, the need for influenza vaccination has not been mentioned in this document as of today, but would be a good opportunity to remind both, physician and pregnant women, about the importance of influenza vaccination during pregnancy.

Half of our participants knew about the existence of the STIKO influenza vaccination recommendation for pregnant women. However, we must assume that this proportion is likely an overestimation of the true knowledge, since many women incorrectly quoted further vaccinations that they should receive during pregnancy. As vaccination appears to be the socially desired answer it seems that such guesses could be biased in the positive direction [21]. However, our results and findings from other studies demonstrated that the correct knowledge of vaccination recommendation is crucial for vaccination uptake [17, 44].

Our study has several limitations: (i) Since our study was an online-based survey we cannot rule out some degree of selection bias. To compare characteristics of study population we used data for women of childbearing age living in Germany since no representative national perinatal dataset exists. It turned out that women born in Germany and women with a higher education were over-represented in our study. If higher education is confounded with higher vaccination knowledge, this could mean a rather overestimation of the presented general vaccination knowledge. Thus, representativeness of the study sample could not be fully determined. However, the vaccination coverage estimate was not substantially different from that identified in a cross-sectional study conducted in Germany that utilized a special sampling frame to reach a more representative sample of the target population [10]. (ii) Due to our recruitment strategy we could not calculate the response rate, since the total number of pregnant women who noticed the online request for study participation is unknown. (iii) Vaccination status was self-reported and can therefore be subject to misclassification. While a recently published study suggested an overestimation for self-reported seasonal influenza vaccination rates compared to vaccination registries [45] other studies revealed an adequate degree of reliability [46, 47]. (iv) We did not ask for underlying chronic diseases, even though they are an indication for receiving the flu shot already during the first trimester of pregnancy in Germany. Therefore, we must assume that approximately 20 % of women stated being vaccinated during their first trimester of pregnancy either were chronically ill, did not know the influenza vaccination recommendation or did not know that they were pregnant when receiving the flu shot. (v) The sample was limited to German-speaking women as the online questionnaire was only available in German. Women who were not able to understand German were therefore not represented in the study population, which might have introduced some selection bias.

## Conclusions

In conclusion, influenza vaccination uptake in pregnant women is low in Germany. Further efforts are needed to promote and educate pregnant women specifically about the benefits and risks of influenza vaccination during pregnancy to protect pregnant women and their infants. Tailored communication strategies for pregnant women should focus especially on improving perceptions related to personal risks regarding the disease and the vaccination. It is concerning, that many pregnant women have a lack of general vaccination knowledge indicating further needs for education about vaccine-related misconceptions. Gynaecologists should be made aware about their crucial role in supporting vaccination decision-making of pregnant women and the need to provide relevant information to counteract misconceptions.
